# Supplementary material for: A trade-off between proliferation and defense in the fungal pathogen Cryptococcus at alkaline pH is controlled by the transcription factor GAT201
Source: bioRxiv. 2023 Jun 14:2023.06.14.543486. Preprint. [Version 1] doi: 10.1101/2023.06.14.543486 (PMC10312749; doi:10.1101/2023.06.14.543486)

bioRxiv preprint doi: https://doi.org/10.1101/2023.06.14.543486; this version posted June 14, 2023. The copyright holder for this preprint (which was not certified by peer review) is the author/funder, who has granted bioRxiv a license to display the preprint in perpetuity. It is made available under aCC-BY 4.0 International license.

Supplementary Figures

Figure S1, related to Figure 1C: Principal Component Analysis of RNA-seq dataset 1. Note PC 1 vs 2 panel is a repeat of 1C.

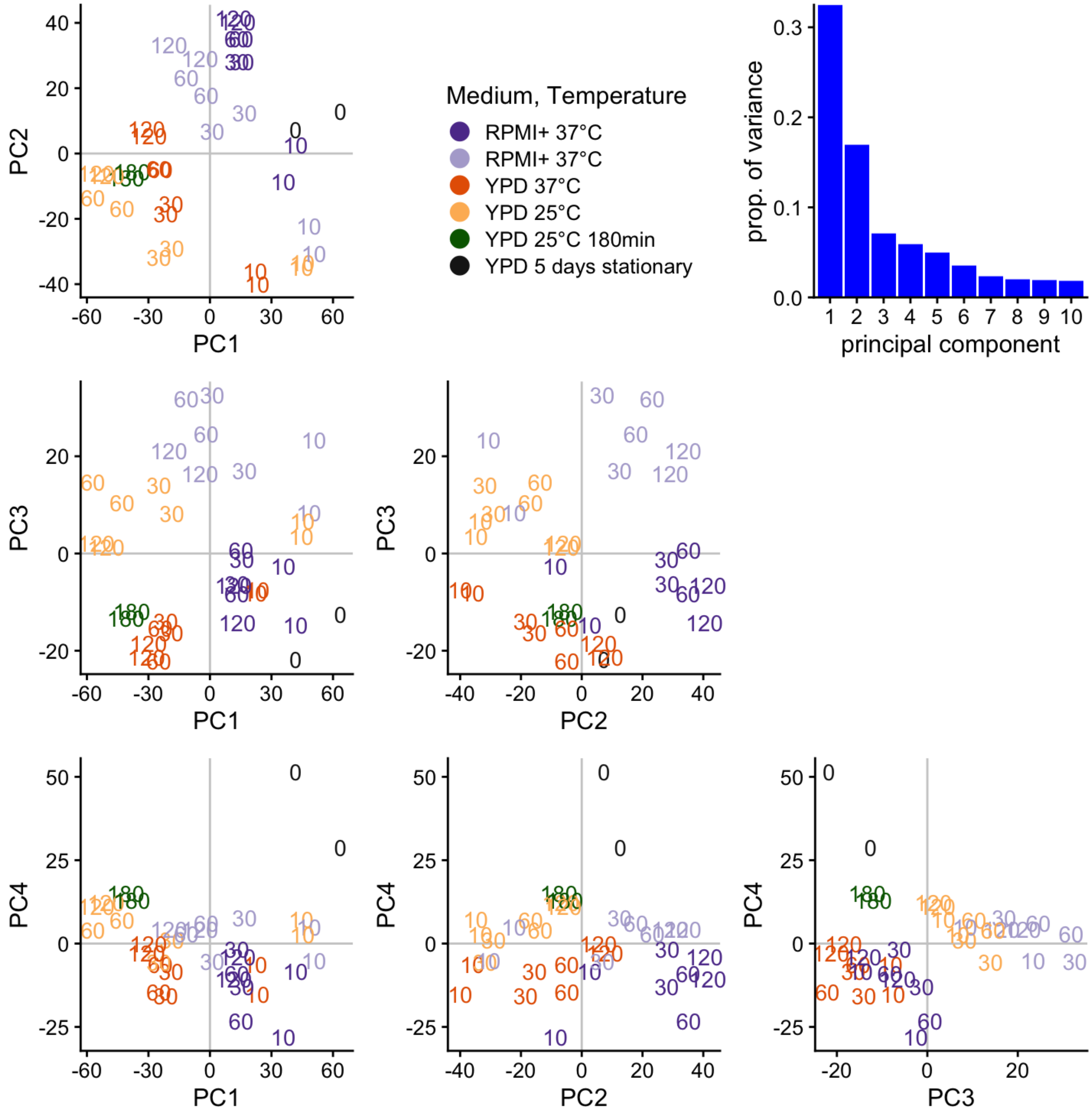

bioRxiv preprint doi: https://doi.org/10.1101/2023.06.14.543486; this version posted June 14, 2023. The copyright holder for this preprint (which was not certified by peer review) is the author/funder, who has granted bioRxiv a license to display the preprint in perpetuity. It is made available under aCC-BY 4.0 International license.

Figure S2, related to Figure 1E: Thirty representative genes show distinct expression patterns, again in log2 fold-change per gene. See Figure 1 legend for details.

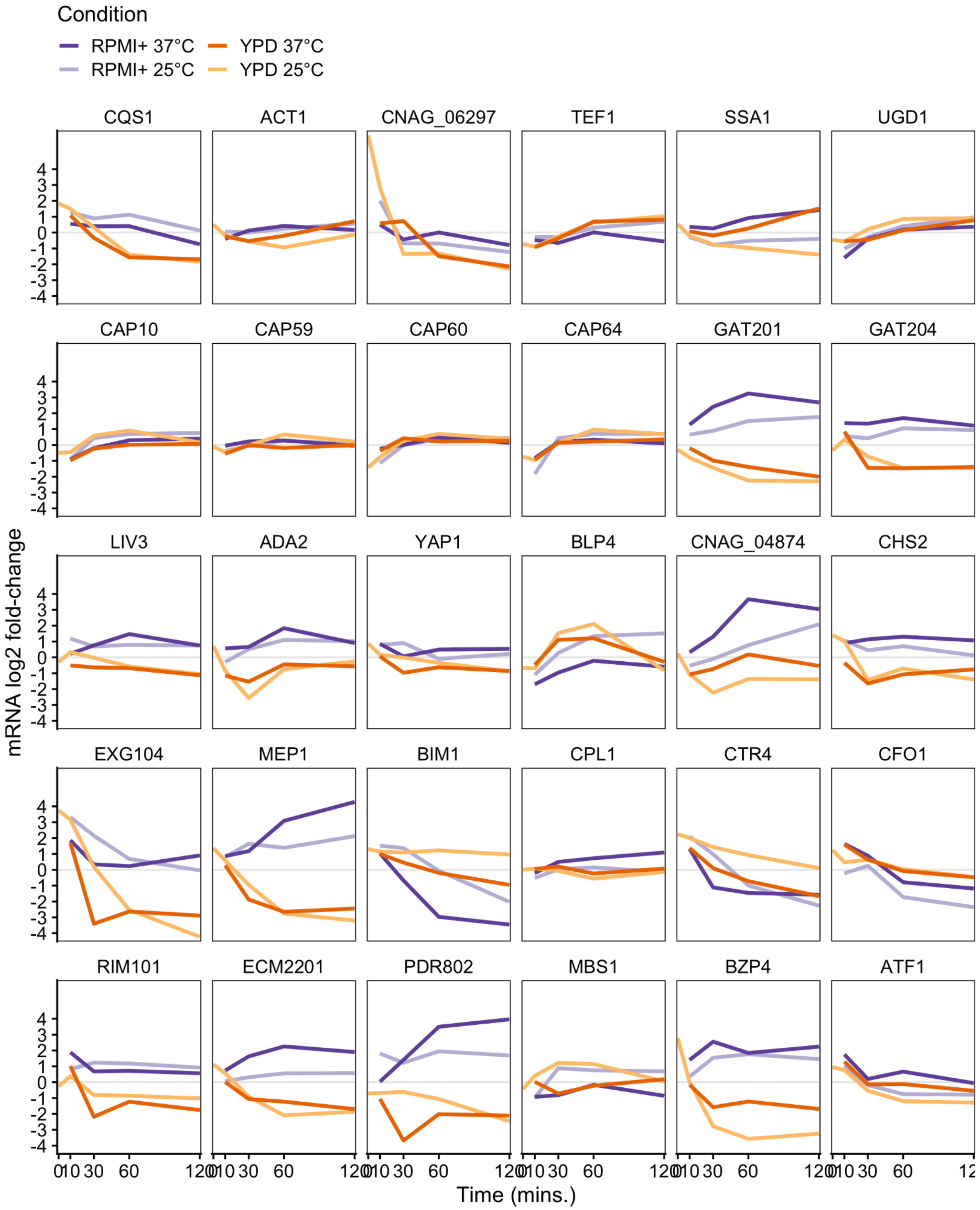

bioRxiv preprint doi: https://doi.org/10.1101/2023.06.14.543486; this version posted June 14, 2023. The copyright holder for this preprint (which was not certified by peer review) is the author/funder, who has granted bioRxiv a license to display the preprint in perpetuity. It is made available under aCC-BY 4.0 International license.

Figure S3, related to figure 2: GAT201 promotes capsule biosynthesis and represses budding in RPMI medium (without serum) at 37°C 2 hours after inoculation. There is no obvious difference in phenotype in rich YPD medium. Micrographs show GAT201 (H99), gat201∆m, and complemented GAT201-C1 strains, stained with India Ink. Scale bar is 10µm.

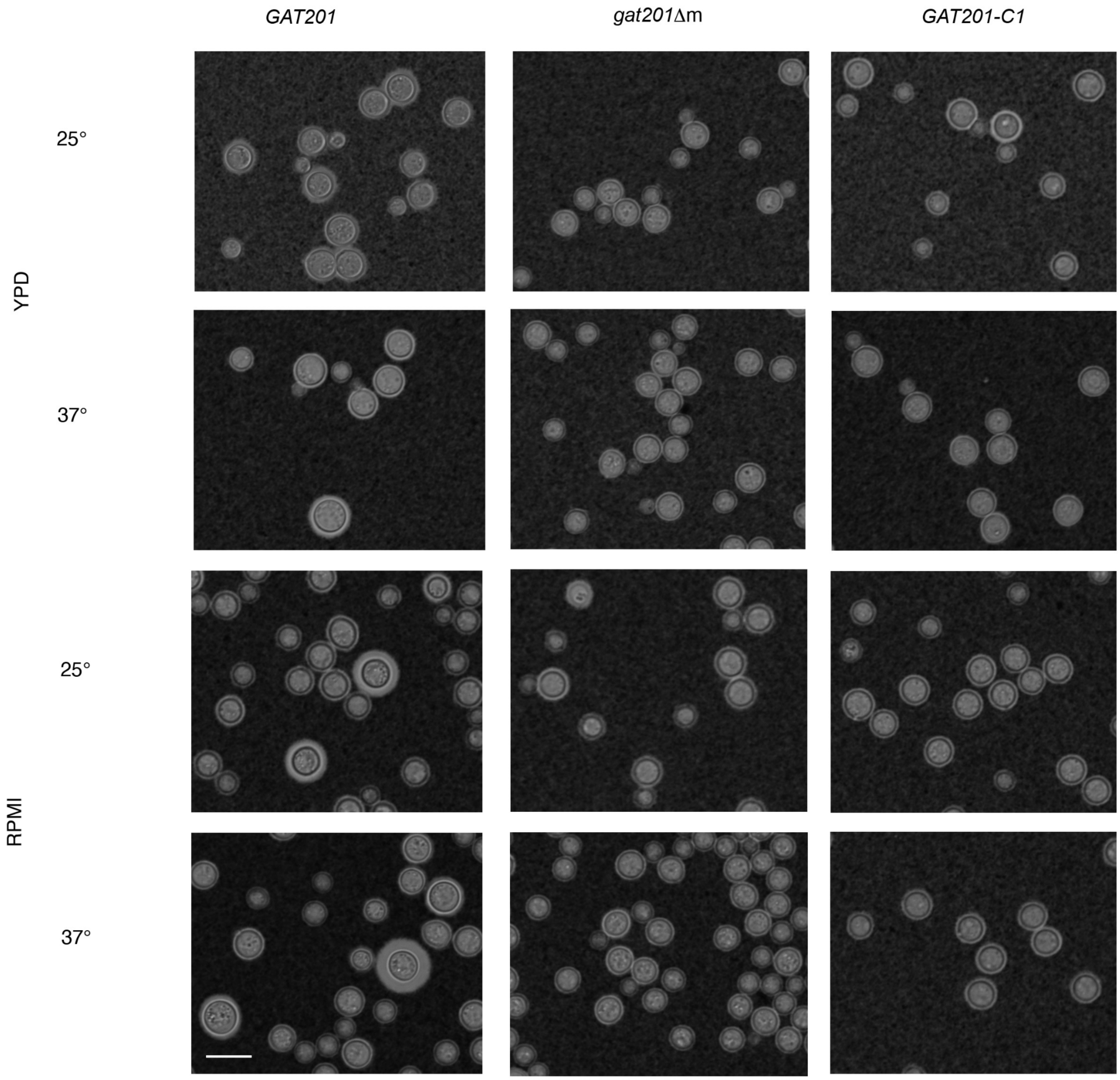

bioRxiv preprint doi: https://doi.org/10.1101/2023.06.14.543486; this version posted June 14, 2023. The copyright holder for this preprint (which was not certified by peer review) is the author/funder, who has granted bioRxiv a license to display the preprint in perpetuity. It is made available under aCC-BY 4.0 International license.

Figure S4, related to Figure 2: Partial complementation of Gat201 mRNA expression shown by RT-qPCR of GAT201, GAT204, and LIV3 genes compared to 3 reference genes (ACT1, GPD1, SRP14). In complemented strains, GAT201 mRNA abundance is roughly 10x lower (log2 fold-change ~3.3) than in wild-type *GAT201* (KN99alpha). Cultures were inoculated from overnight growth in YPD and grown in RPMI at 37°C for 7 hours. The figure shows log2 fold-change (ΔΔCq) values from 3 biological replicates (median of 3 technical replicates), and a mean value across the biological replicates. The low value of GAT201 detected in *gat201Δm* represents background, and was only detected at all in 2 out of 3 biological replicates.

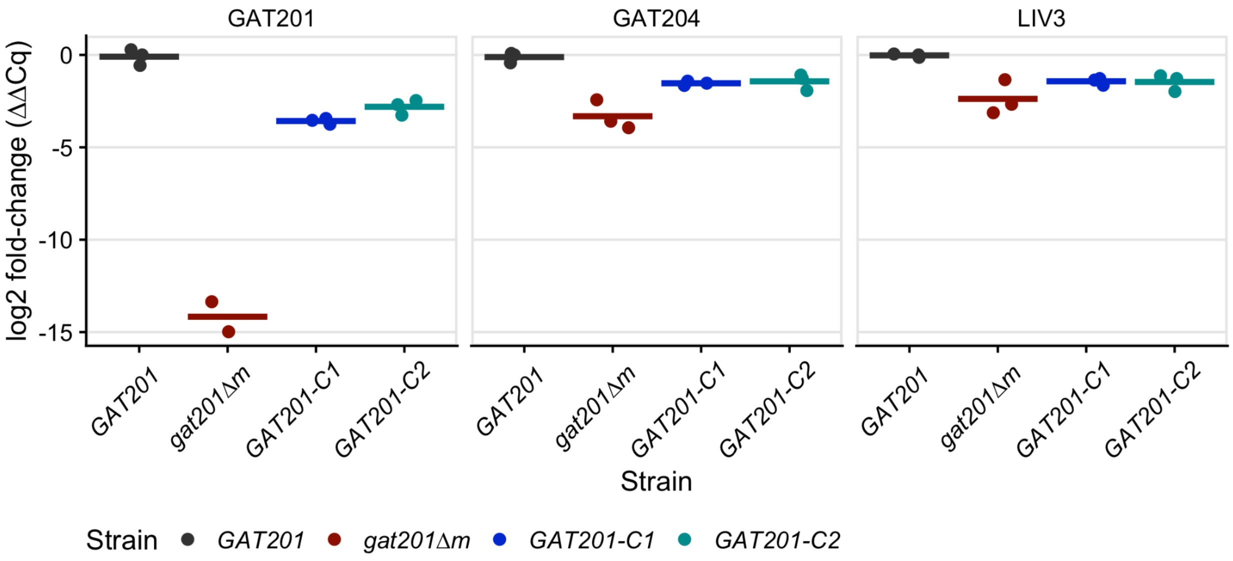

bioRxiv preprint doi: https://doi.org/10.1101/2023.06.14.543486; this version posted June 14, 2023. The copyright holder for this preprint (which was not certified by peer review) is the author/funder, who has granted bioRxiv a license to display the preprint in perpetuity. It is made available under aCC-BY 4.0 International license.

Figure S5: GAT201 has a minor effect on growth in media buffered to near-neutral pH with mono and dibasic sodium phosphate and β-glycerophosphate. Cells were grown as in Figure 2C and their OD595 measured, but in Gibco™ CO_2-independent media.

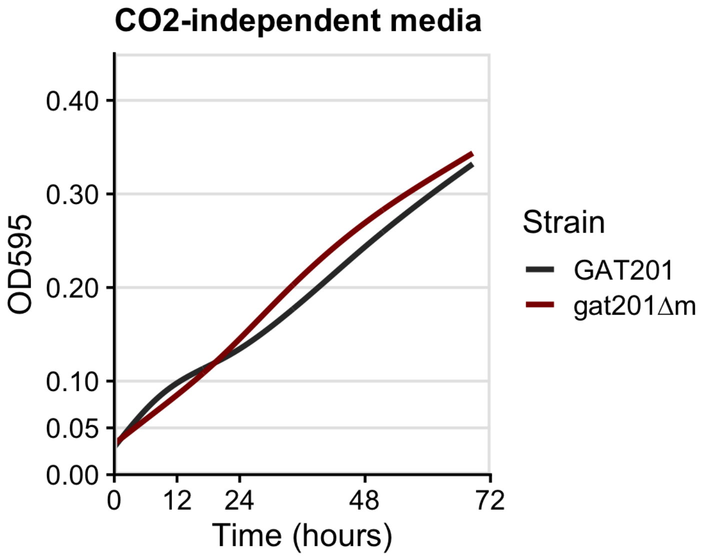

bioRxiv preprint doi: https://doi.org/10.1101/2023.06.14.543486; this version posted June 14, 2023. The copyright holder for this preprint (which was not certified by peer review) is the author/funder, who has granted bioRxiv a license to display the preprint in perpetuity. It is made available under aCC-BY 4.0 International license.

Figure S6, related to Figure 3A: *GAT201* promotes capsule biosynthesis and represses budding in RPMI medium both with and without serum at 37°C, 2 hours after inoculation. Strains are *GAT201* (KN99alpha) and *gat201*Δ*m*, here a wider field of view is shown than in Figure 3A.

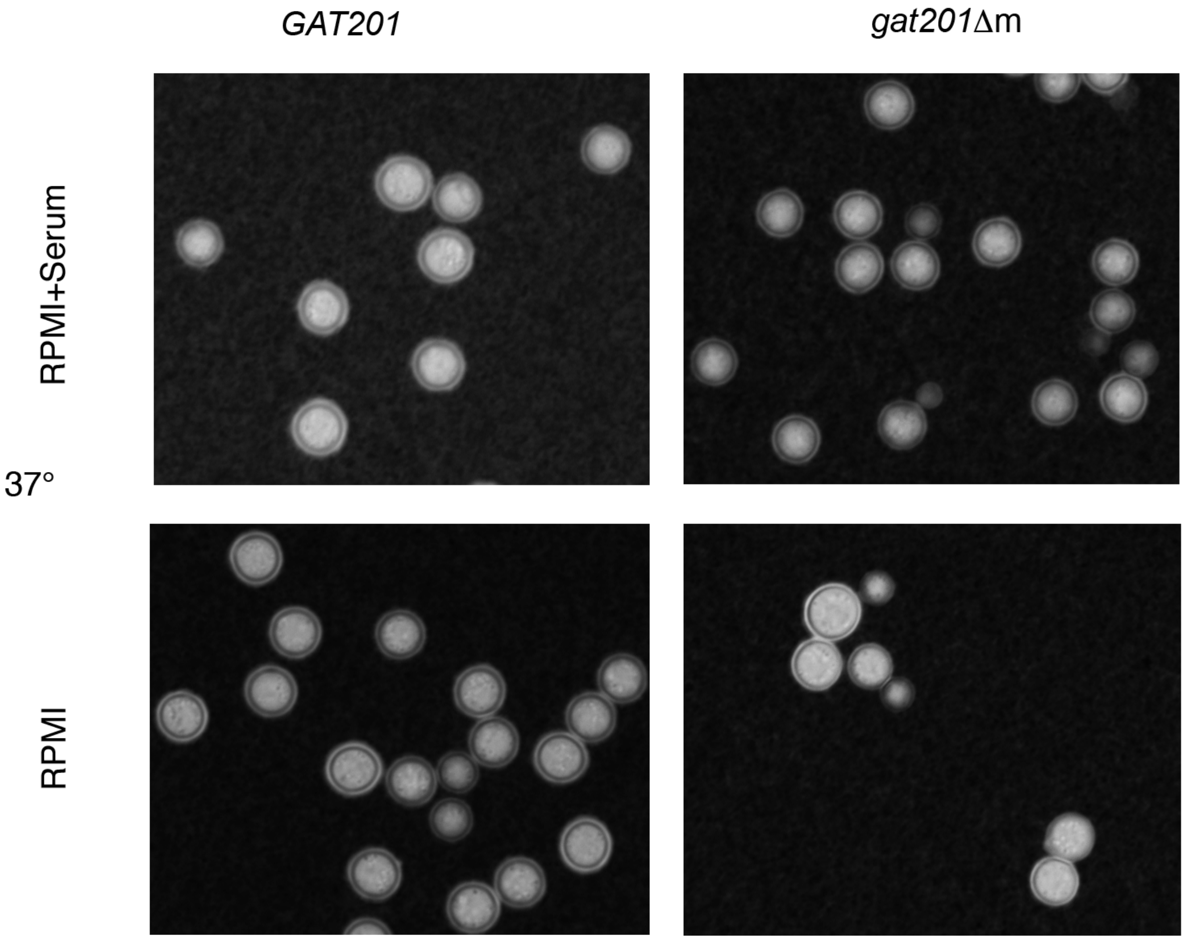

bioRxiv preprint doi: https://doi.org/10.1101/2023.06.14.543486; this version posted June 14, 2023. The copyright holder for this preprint (which was not certified by peer review) is the author/funder, who has granted bioRxiv a license to display the preprint in perpetuity. It is made available under aCC-BY 4.0 International license.

Figure S7, related to Figure 3B: Principal Component Analysis of RNA-seq dataset 2, comparing *GAT201* (wild-type) to *gat201*Δ strains. Note PC 1 vs 2 panel is a repeat of 3B.

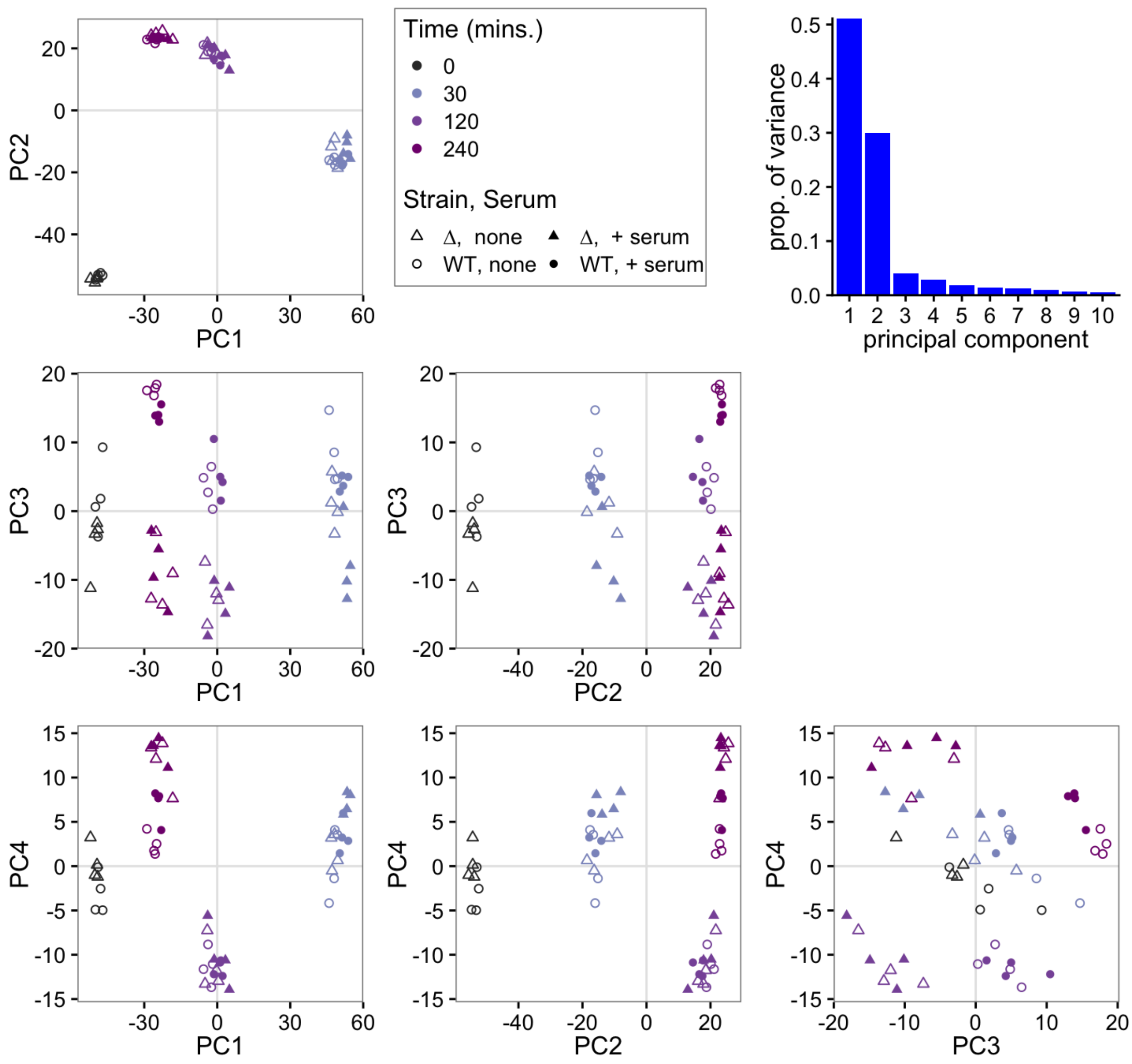

bioRxiv preprint doi: https://doi.org/10.1101/2023.06.14.543486; this version posted June 14, 2023. The copyright holder for this preprint (which was not certified by peer review) is the author/funder, who has granted bioRxiv a license to display the preprint in perpetuity. It is made available under aCC-BY 4.0 International license.

Figure S8, related to Figure 3D: Thirty representative genes show distinct expression patterns and GAT201 dependence, again in log2 fold-change per gene. See Figure 3 legend for details.

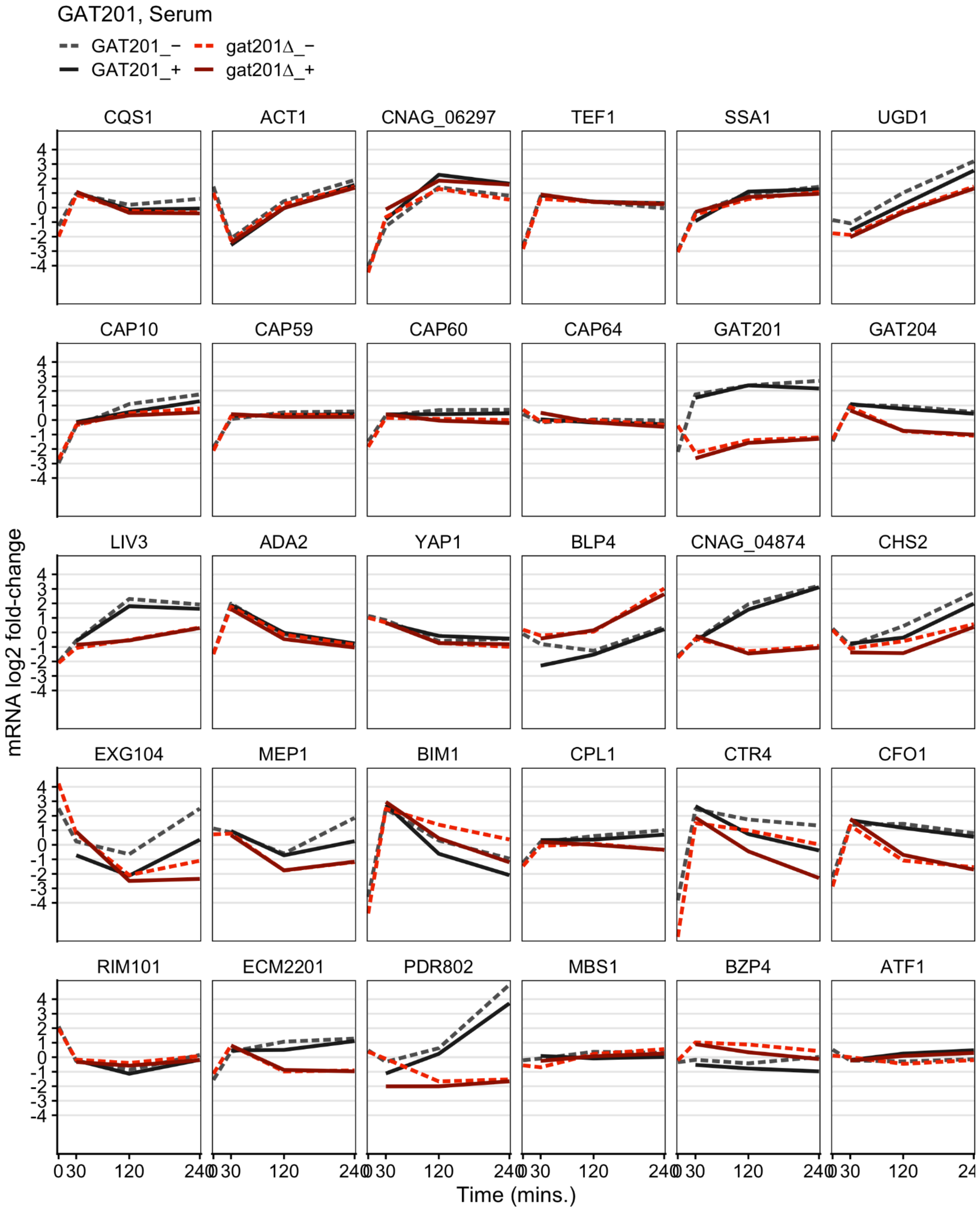

bioRxiv preprint doi: https://doi.org/10.1101/2023.06.14.543486; this version posted June 14, 2023. The copyright holder for this preprint (which was not certified by peer review) is the author/funder, who has granted bioRxiv a license to display the preprint in perpetuity. It is made available under aCC-BY 4.0 International license.

bioRxiv preprint doi: https://doi.org/10.1101/2023.06.14.543486; this version posted June 14, 2023. The copyright holder for this preprint (which was not certified by peer review) is the author/funder, who has granted bioRxiv a license to display the preprint in perpetuity. It is made available under aCC-BY 4.0 International license.

Figure S9, related to Figure 3. Serum affects expression of a small set of transcripts. A, Number of differentially expressed genes (2x changed, 5% FDR) dependent on serum at each timepoint in *GAT201* and *gat201*Δ strains. B, Serum-dependent log2 fold-change and p-values in *GAT201* at 4 hours. C, Serum-dependent log2 fold-change and p-values in *gat201*Δ at 4 hours.

bioRxiv preprint doi: https://doi.org/10.1101/2023.06.14.543486; this version posted June 14, 2023. The copyright holder for this preprint (which was not certified by peer review) is the author/funder, who has granted bioRxiv a license to display the preprint in perpetuity. It is made available under aCC-BY 4.0 International license.

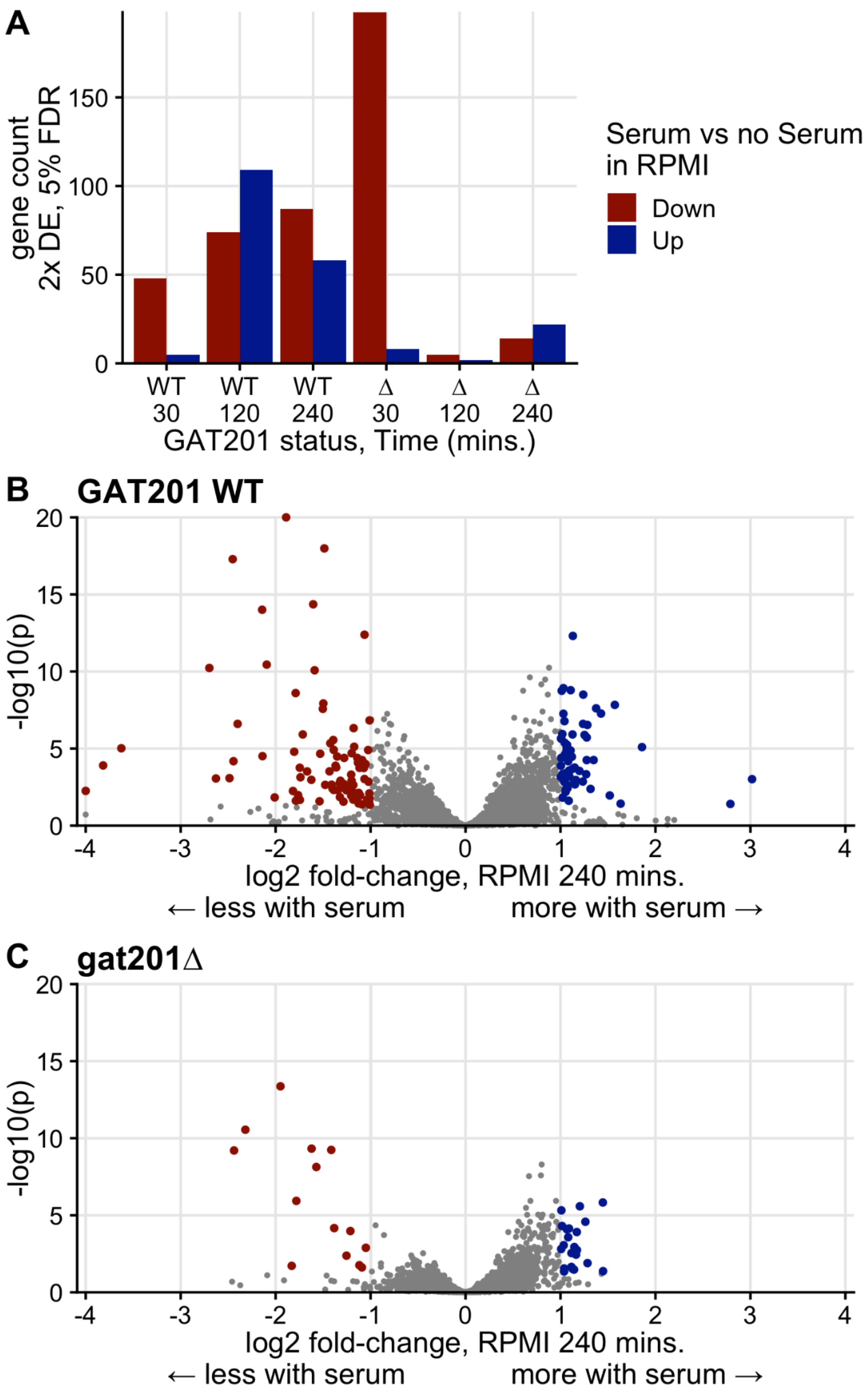

bioRxiv preprint doi: https://doi.org/10.1101/2023.06.14.543486; this version posted June 14, 2023. The copyright holder for this preprint (which was not certified by peer review) is the author/funder, who has granted bioRxiv a license to display the preprint in perpetuity. It is made available under aCC-BY 4.0 International license.

Figure S10: Comparison of differential expression in dataset 1 (Wakeup) and dataset 2 (Gat201), differential expression between 0 hours (pre-inoculation timepoint) and 2 hours in RPMI + serum for wild-type strain. The lowest 10% expressed transcripts in both of these datasets (baseMean from DESeq2) were filtered out. 30 genes shown in Figures S2, S8 and discussed in the text are highlighted.

bioRxiv preprint doi: https://doi.org/10.1101/2023.06.14.543486; this version posted June 14, 2023. The copyright holder for this preprint (which was not certified by peer review) is the author/funder, who has granted bioRxiv a license to display the preprint in perpetuity. It is made available under aCC-BY 4.0 International license.

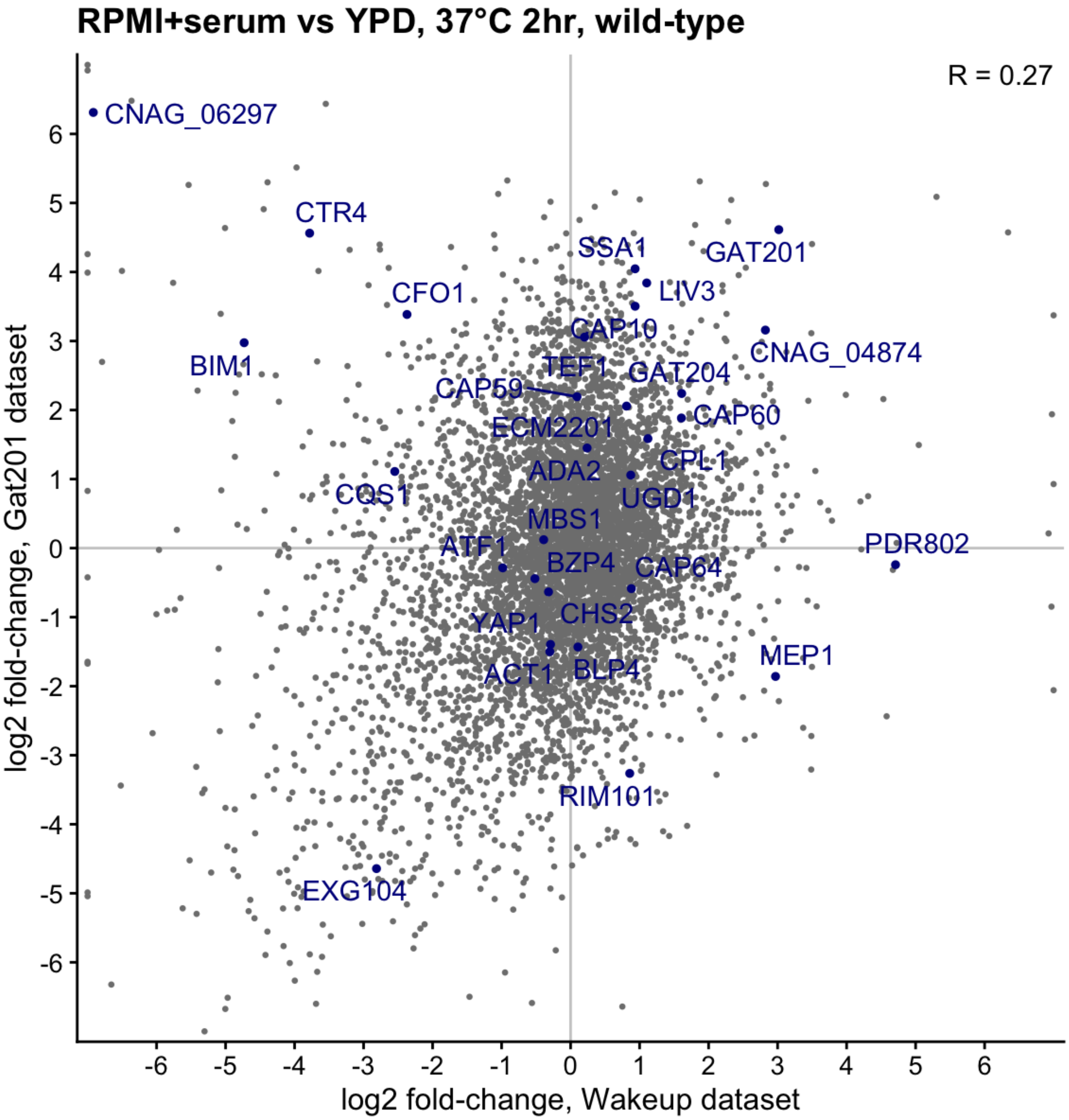

bioRxiv preprint doi: https://doi.org/10.1101/2023.06.14.543486; this version posted June 14, 2023. The copyright holder for this preprint (which was not certified by peer review) is the author/funder, who has granted bioRxiv a license to display the preprint in perpetuity. It is made available under aCC-BY 4.0 International license.

Figure S11: Growth curves of mutants in the Gat201 pathway. Panels A,B,C show growth curves, all 4 biological replicates in 3 technical replicates, of OD595 corrected for values of blank wells. Individual wells are plotted as faint lines, and smoothing spline for each strain plotted as thick lines.

bioRxiv preprint doi: https://doi.org/10.1101/2023.06.14.543486; this version posted June 14, 2023. The copyright holder for this preprint (which was not certified by peer review) is the author/funder, who has granted bioRxiv a license to display the preprint in perpetuity. It is made available under aCC-BY 4.0 International license.

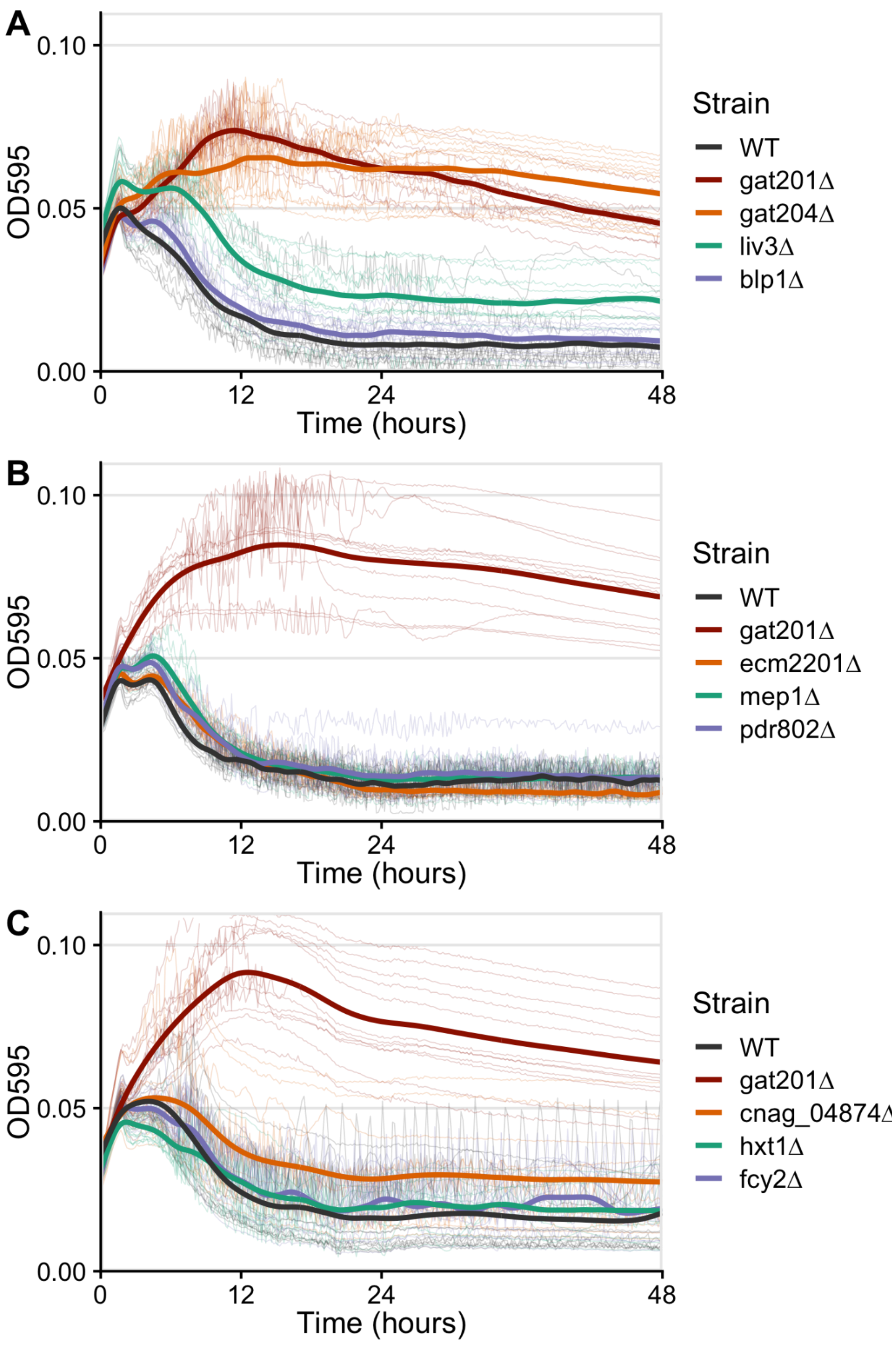

Supplement: Supplementary file 1 [file NIHPP2023.06.14.543486v1-supplement-4.pdf]
